# A Chinese Herbal Decoction, Modified Yi Guan Jian, Induces Apoptosis in Hepatic Stellate Cells through an ROS-Mediated Mitochondrial/Caspase Pathway

**DOI:** 10.1155/2011/459531

**Published:** 2010-09-26

**Authors:** Hung-Jen Lin, Ching-Ping Tseng, Chia-Fan Lin, Mei-Huei Liao, Chuan-Mu Chen, Shung-Te Kao, Ju-Chien Cheng

**Affiliations:** ^1^School of Chinese Medicine, College of Chinese Medicine, China Medical University, Taichung 404, Taiwan; ^2^Department of Medical Biotechnology and Laboratory Science, Chang Gung University, Taoyuan 333, Taiwan; ^3^Department of Medical Laboratory Science and Biotechnology, China Medical University, Taichung 404, Taiwan; ^4^Department of Life Sciences, National Chung Hsing University, Taichung 402, Taiwan

## Abstract

The Chinese herb modified Yi Guan Jian (mYGJ) is an effective regimen that is usually used in outpatients with chronic liver diseases such as fibrosis and cirrhosis. However, the mechanism for the action of mYGJ on liver fibrosis is not yet clear. In this study, we found that mYGJ induced hepatic stellate cells (HSCs) apoptosis concomitant with the downregulation of Bcl-2 expression and slight elevation of Bax level. Moreover, the reactive oxygen species (ROS) were generated in the early stages of mYGJ-induced HSCs apoptosis to facilitate calcium and cytochrome c release from the mitochondria to cytosol. Subsequently, caspase 9 and caspase 3 were activated. Furthermore, the activation of ER stress-associated caspase 12 in HSCs was also evaluated. Together, we report the first evidence-based study to demonstrate that mYGJ decoction induces HSCs apoptosis through ROS accumulation and the intrinsic apoptosis pathway. These findings provide rationale for further clinical investigation of traditional Chinese medicine recipes against liver fibrosis.

## 1. Introduction

Liver fibrosis is caused by severe liver damage that occurs in many patients with liver injury such as persistent viral and helminthic infections, overuse of alcohol or nonalcoholic steatohepatitis (NASH), autoimmunity, drug intoxication, and some hereditary disease [1, 2]. Hepatic stellate cell (HSC) proliferation and activation are thought to be crucial for the progression of liver fibrosis [3]; HSCs change from a quiescent status to activation and undergo transformation into myofibroblast-like cells which produce excessive extracellular matrix (ECM) proteins including *α*-smooth muscle actin (*α*-SMA) and type I collagen. Excessive depositions of these ECM proteins in liver tissue result in fibrogenesis and disturb matrix degradation that causes the loss of homeostasis in liver tissue followed by progression to liver cirrhosis and hepatocellular carcinoma [4, 5]. 

Recent studies reveal that liver fibrosis and cirrhosis is a reversible process [6, 7]. Removal of the liver injury-causing factors followed by a decrease in proinflammatory cytokines and an induction of activated HSC apoptosis may cause liver fibrosis diminution [8]. Therefore, suppression of HSC activation and proliferation and induction of apoptosis in activated HSCs have been proposed as therapeutic strategies for the treatment and prevention of hepatic fibrosis [1, 9, 10]. 

Yi Guan Jian (YGJ) decoction consists of six kinds of Chinese herbs and is a traditional Chinese hepato-therapeutic herbal formula. According to the Chinese medicine theory, the modified Yi Guan Jian (mYGJ) is formulated by adding three more herbs including *Astragalus membranaceus*, *Trionyx sinensis* Wiegmann (Carapax Trionycis), and *Eupolyphaga sinensis* Walker into the original YGJ to activate blood and resolve stasis, tonifies qi, and hard mass for the patients with liver diseases. Although there is still no evidence-based clinical study, YGJ has shown apparent efficacy in outpatients with chronic liver diseases such as fibrosis and cirrhosis to improve clinical symptoms, liver function, and quality of life for patients. Besides, the major active components of YGJ extract, ferulic acid and catalpol, significantly inhibit the progression of hepatic fibrosis in carbon tetrachloride-induced animal model study [11]. However, the underlying mechanism for the antifibrotic effects of YGJ or mYGJ is still unclear. In this study, we demonstrate that mYGJ inhibits HSC-T6 hepatic stellate cell proliferation concomitant with a significant decrease in the expression of liver fibrosis marker *α*-SMA. This effect is mediated by induction of HSCs apoptosis through an ROS-mediated mitochondrial/caspase signaling pathway. These findings thereby show for the first time the experimental evidence for the antifibrotic effects of mYGJ.

## 2. Methods

### 2.1. Materials

The anti-*α*-SMA, anti-*β*-actin, and catalase were purchased from Sigma (Saint Louis, MO). The anti-Bcl-2, anti-Bax, and anti-GRP78 antibodies were purchased from Santa Cruz Biotechnology (Santa Cruz, CA). The anticaspase 3, anticleaved caspase 9, and anticalpain antibodies were purchased from Cell Signaling Technology (Beverly, MA). The Mitochondria/Cytosol Fractionation Kit and the anticytochrome c antibody were purchased from BioVision (Mountain View, CA). The Fluorescein FragEL DNA Fragmentation Detection Kit was purchased from EMD Chemicals (Gibbstown, NJ). The 3-(4, 5-dimethylthiazol-2-yl)-5-(3-carboxymethoxyphenyl)-2-(4-sulfophenyl)-2H-tetrazolium (MTS) assay kit was purchased from Promega (Madison, WI).

### 2.2. Preperation of Modified Yi Guan Jian

The mYGJ extract was kindly provided by Ko-Da Pharmaceutical Company, Taiwan. The composition of mYGJ was listed in Table 1 and the index constituents were shown in the Supplemental Figure 1. The mYGJ liquid extracts were composed of Glehnia littoralis F., Ophiopogon japonicus (L. f.) Ker-Gawl., Angelica sinensis (Oliv.) Diels., Rehmannia glutinosa Libosch., Lycium barbarum L., Melia toosendan Sieb. et Zucc., Astragalus membranaceus (Fisch.) Bge., Trionyx sinensis Wiegmann., and Eupolyphaga sinensis Walker. The three components of modified YGJ that were different from original YGJ were listed in Table 1 with bold face. The concentration for the final stock solution of mYGJ extract was 175 mg/mL.

### 2.3. Cell Culture and Proliferation Assay

The immortalized rat myofibroblast cell line HSC-T6 was a kind gift of Dr. Scott L. Friedman (Mount Sinai School of Medicine, New York, NY). The cells were cultured in Dulbecco's Modified Eagle's Medium (DMEM) supplemented with 10% fetal bovine serum at 37°C in a humidified atmosphere of 5% CO_2_. For cell proliferation assay, the HSC-T6 cells were seeded into a 96-well culture plate at a cell density of 1 × 10^4^ cells/well for 24 hours. Then, the cells were treated with a serial concentration of mYGJ for the indicated time and cell proliferation was determined by MTS assay as described by the manufacture (Promega).

### 2.4. Western Blot Analysis

The cell lysates were harvested in lysis buffer (50 mM HEPES, pH 7.5, 150 mM NaCl, 1% Triton X-100, 1.5 mM MgCl_2_, 10% glycerol, protease inhibitor cocktail (Invitrogen)) and were separated on a 10% SDS-polyacrylamide gel as described previously [12]. For cellular fractionation of cytochrome c, the cytosolic and the mitochondria fractions of the lysates were obtained using the Mitochondria/Cytosol Fractionation Kit according to the manufacturer's (BioVision) instruction. Western blot analysis was then performed as described in [13].

### 2.5. TUNEL Assay

Terminal deoxynucleotidyl transferase (TdT) dUTP nick end labeling (TUNEL) was performed by the Fluorescein FragEL DNA Fragmentation Detection Kit according to the manufacturer's (EMD Chemicals) instruction. Briefly, HSC-T6 cells (1 × 10^6^ cells/35-mm culture dish) were treated with the indicated dose of mYGJ. At 72 hours after treatment, the cells were fixed with 4% paraformaldehyde at room temperature for 30 minutes. The fixed cells were washed with phosphate-buffered saline (PBS) and then cytospun to transfer cells to a glass slide. The cells were then treated with proteinase K (2 *μ*g/mL) at room temperature for 5 minutes. After adding 100 *μ*L of TdT equilibration buffer for 30 minutes, the cells were incubated with 60 *μ*L of TdT Labeling Reaction Mix (containing 3 *μ*L TdT) at 37°C for 1 hour. Following several washes with Tris-buffered saline (TBS), the stained cells were observed by fluorescence microscopy (OLYMPUS, IX70).

### 2.6. Measurements of Reactive Oxygen Species (ROS) and Cytosolic Calcium Concentration

For measurements of ROS accumulation and quantification, the cells were incubated with medium containing 1 *μ*M H_2_DCFDA (Molecular probes) in dark at 37°C for 30 minutes. The cells were trypsinized, washed by PBS, and then analyzed immediately by flow cytometry using FACS Calibur (Becton Dickinson). For catalase compensation assay, the catalase (600 U/mL) was added into culture media for 2 hours before incubation with H_2_DCFDA.

For measurement of cytosolic calcium concentration, the cells were treated with 1 *μ*M Fluo-4/AM (BIOCHEMIKA) in dark at room temperature for 30 minutes. After washing twice with 1X PBS, the cells were subjected to flow cytometric analysis.

### 2.7. Statistical Analysis

Data were expressed as mean ± standard deviation (SD) or mean ± standard error (SE). Statistical analysis was performed by Student's *t*-test. A *P* < .05 was considered as statistically significant.

## 3. Results

### 3.1. mYGJ Induces HSC-T6 Cell Apoptosis and Inhibits Liver Fibrosis Marker Protein

**Figure 1:**
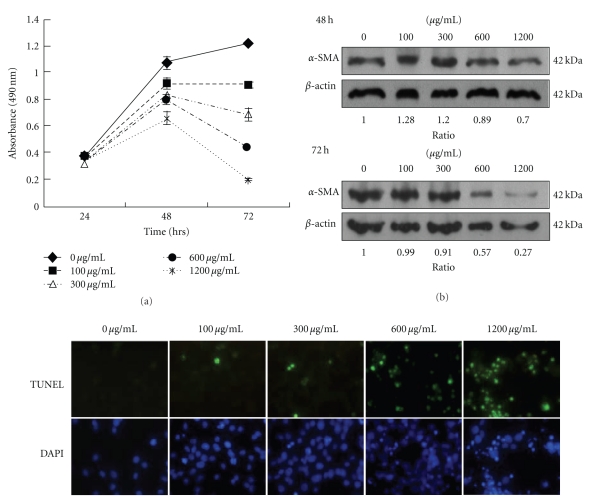
The mYGJ exhibits potent antifibrosis activity on HSC-T6 cells. (a) HSC-T6 cells were seeded onto 96-well plates and the indicated dose of mYGJ was added to the medium for 24, 48, and 72 hours, respectively. Cell viability was measured by MTS assay as described in the Material and methods. The data represented the mean ± S.D. of three independent experiments. (b) HSC-T6 cells were treated with the indicated concentration of mYGJ. The cell lysates were harvested at 48 and 72 hours posttreatment and were subjected to Western blot analysis using the anti-*α*-SMA antibody. The expression of *β*-actin was used for the control of equal protein loading. The band intensity of Bcl-2 or Bax versus *β*-actin was determined and the relative ratio to control experiment which did not treat with mYGJ was indicated below the data. (c) Representative photographs of HSC-T6 cells that were treated with the indicated concentrations of mYGJ for 72 hours. The determination of apoptosis was performed by TUNEL assay with the apoptotic cells appeared with green fluorescence. Nuclei were counter-stained using DAPI (blue).

In this study, we used HSC-T6 cells to evaluate whether the traditional Chinese medicine decoction mYGJ elicits antiliver fibrosis activity. At first, the HSC-T6 cells were treated with various concentrations of mYGJ. The cell viability and cell growth were determined by MTS assay. The data indicated that mYGJ inhibited cell survival in a dose-dependent manner (Figure 1(a)). The concentration required for 50% inhibition of growth (IC50) was about 300 *μ*g/mL after 72 hours treatment (*n* = 3). Concomitant with the decrease in cell growth, expression of the stellate cell activation marker protein *α*-SMA was also inhibited by mYGJ (Figure 1(b)). These data indicate that mYGJ inhibits HSC-T6 hepatic stellate cell proliferation concomitant with a decrease in the expression of liver fibrosis marker *α*-SMA.

To determine whether apoptosis is the underlying mechanism for mYGJ to suppress stellate cell growth, HSC-T6 cells were treated with various concentrations of mYGJ for 72 hours and were subjected to TUNEL staining using fluorecein-labeled dUTP for assessment of the degree of apoptosis. As shown in Figure 1(c), the number of TUNEL-positive cells was increased proportional to the increase in the dosage of mYGJ. The apoptotic index as defined by the percentage of cells with green fluorescent signal was 0%, 9%, 11%, 52%, and 80% for the control HSC-T6 cells and the cells treated with mYGJ at the concentration of 100 *μ*g/mL, 300 *μ*g/mL, 600 *μ*g/mL, and 1200 *μ*g/mL, respectively, (Figure 1(c)). These data indicate that mYGJ induces HSC-T6 cell apoptosis in a dose-dependent manner.

### 3.2. mYGJ Enhances ROS Accumulation and Facilitates Calcium and Cytochrome c Release

**Figure 2:**
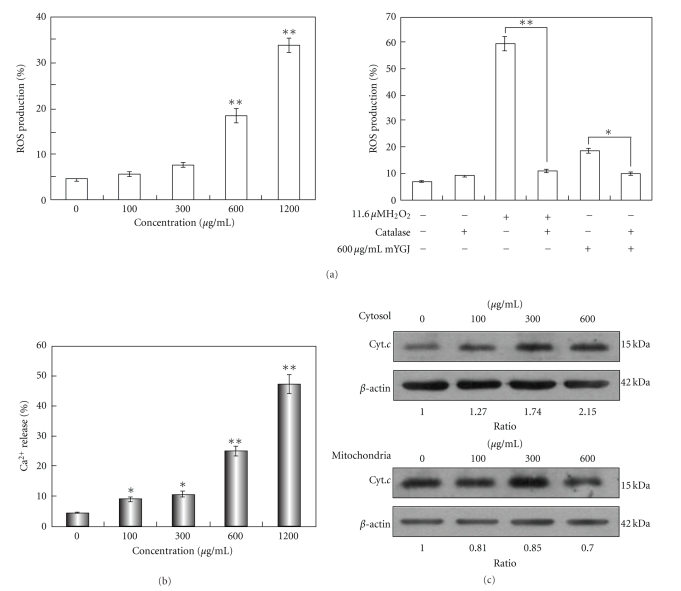
mYGJ enhances ROS accumulation and facilitates calcium and cytochrome *c* release (a) HSC-T6 cells were treated with the indicated concentrations of mYGJ for 24 hours and production of ROS was analyzed by percentage of H_2_DCFDA-stained cells which were determined by flow cytometry (left). Moreover, the HSC-T6 cells that were treated with 600 *μ*g/mL of mYGJ were preincubated with catalase (600 U/mL) 2 hours before ROS measurement by flow cytometry. H_2_O_2_ was used as positive control for the experiment (right). The data represented the mean ± S.E. of three independent experiments. (b) HSC-T6 cells were treated with the indicated concentrations of mYGJ for 48 hours and calcium release was measured by percentage of Fluo-4/AM-stained cells which were determined by flow cytometry. The values *P < .05 and **P < .01 when compared with control cells were considered statistically significant by the paired sample *t*-test. The zero concentration was defined as control. (c) HSC-T6 cells were treated with the indicated concentrations of mYGJ. The cell lysates were harvested at 72 hours post-treatment followed by subfractionation into mitochondria and cytosolic fraction. Western blot analysis was then performed using the anticytochrome *c* antibody. The expression of *β*-actin was used for the control of equal protein loading. The band intensity of Bcl-2 or Bax versus *β*-actin was determined and the relative ratio to control experiment which did not treat with mYGJ was indicated below the data.

One component of mYGJ, *Melia toosendan *Sieb, was reported to induce primary hepatocyte death process by generation of ROS and MAP kinases activation [14]. To delineate ROS generation accounts for the underlying mechanisms of mYGJ-mediated HSCs apoptosis, the HSC-T6 cells were treated with various concentrations of mYGJ and the levels of ROS generation were measured. As shown in left panel of Figure 2(a), ROS generation was increased in a dose-dependent manner. The mYGJ- (600 *μ*g/mL) induced ROS accumulation can be partially reversed when HSC-T6 cells were pretreated with catalase (600 U/mL) for 2 hours (Figure 2(a), right panel). These results suggest that mYGJ enhances ROS accumulation leading to apoptosis of HSCs. 

To reveal whether ROS-mitochondrial signaling is involved in mYGJ-induced apoptosis, HSC-T6 cells were treated with various concentrations of mYGJ and the cytosolic concentration of calcium and cytochrome *c* was measured. By staining the cells with Fluo-4/AM followed by flow-cytometry analysis, our data revealed that mYGJ caused an increase in the concentration of cytosolic calcium in a dose-dependent manner of mYGJ (Figure 2(b)). On the other hand, Western blot analysis of the cytosol and mitochondria fraction of the mYGJ-treated HSC-T6 cell lysates revealed an increase in cytosolic cytochrome *c* and a decrease in mitochondrial cytochrome *c* in a dose-dependent manner of mYGJ (Figure 2(c)). These data indicate that the mYGJ-induced HSCs apoptosis is mediated by the mitochondria-dependent pathway.

### 3.3. mYGJ Induces HSC-T6 Cell Apoptosis Through a Caspase-Dependent Pathway

**Figure 3:**
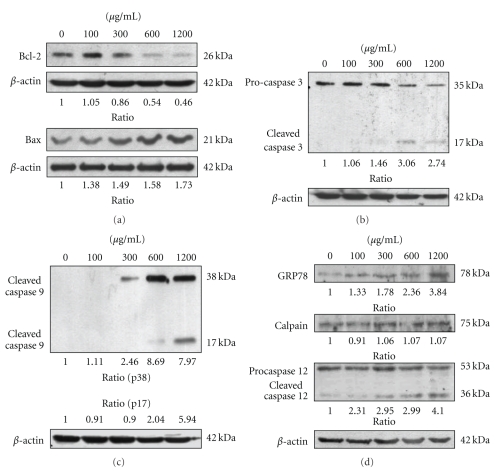
The mYGJ induces HSC-T6 cell apoptosis through a caspase-dependent pathway. (a) HSC-T6 cells were treated with the indicated dose of mYGJ. The cell lysates were harvested at 72 hours post-treatment and were subjected to Western blot analysis using the anti-Bcl-2 and anti-Bax antibodies, respectively. The band intensity of Bcl-2 or Bax versus *β*-actin was determined. (b) and (c) HSC-T6 cells were treated with the indicated concentrations of mYGJ. The cell lysates were harvested at 72 hours post-treatment and were subjected to Western blot analysis using the anticaspase-3 (b) and anticaspase 9 (c) antibodies, respectively. The band intensity of cleaved caspase 3 or cleaved caspase 9 versus *β*-actin was determined. (d) HSC-T6 cells were treated with the indicated concentrations of mYGJ. The cell lysates were harvested at 72 hours post-treatment and were subjected to Western blot analysis using the anti-GRP78, anti-calpain, and anti-caspase 12 antibodies, respectively. The band intensity of cleaved caspase-3 or cleaved caspase 9 versus *β*-actin was determined. The expression of *β*-actin was used for the control of equal protein loading and the relative band intensity ratio to control experiment which did not treat with mYGJ was indicated below the data.

In the intrinsic pathway, death signals act directly or indirectly on the mitochondria, resulting in the release of cytochrome *c*. Members of the B-cell lymphoma protein-2 (Bcl-2) family play an important role in regulation of cytochrome *c* release and cytochrome *c*-mediated apoptosis [15]. To validate mYGJ induced-cytochrome *c* release in HSC-T6 cells was controlled by proteins of Bcl-2 family, the antiapoptotic factor Bcl-2 and proapoptotic factor Bax expression were analyzed in mYGJ-treated HSC-T6 cells. Our data indicated that mYGJ resulted in a decrease in Bcl-2 and a moderate increase in Bax (Figure 3(a)).

Caspase activation is the key event in apoptotic cell death. To further verify that mYGJ-induced apoptotic death in HSC-T6 cells, the activation status of caspase 3, 8, and 9 in mYGJ-treated HSC-T6 was measured by Western blot analysis. The active and cleavage forms of caspase 3 and 9 were observed in the lysates of HSC-T6 cells that were treated with 300, 600, and 1200 *μ*g/mL of mYGJ (Figures 3(b) and 3(c)). In contrast, no active form of caspase 8 was observed in these cell lysates (data not shown). 

Alteration of intracellular calcium homeostasis plays an important role in initiating the apoptotic response. It may be promoted by ROS accumulation or from endoplasmic reticulum (ER) stress. Calpain and caspase 12 are the two major ER-related cell death signaling molecules, while GRP-78 is a well known marker of ER stress. To determine whether the calcium release-activated apoptosis of mYGJ-treated HST-6 cells was associated with ER stress, the cleavage of caspase 12 was analyzed by Western blot analysis. Our data revealed that mYGJ treatment increased caspase 12 cleavage concomitant with the increase in the expression of GRP-78 but not calpain (Figure 3(d)). Together, these results suggest that mYGJ induces ER stress and caspase 12 activation following caspase 9 and 3 activation leading to cell death of HSC-T6.

## 4. Discussion

Traditional Chinese medicine has been used in China for thousands of years. Recipes for treatment of liver disease such as liver fibrosis have been demonstrated to improve clinical management of patients. Here, we report the first evidence-based study to elucidate that mYGJ elicits its antifibrotic activity through inhibition of HSCs activation and downregulation of *α*-SMA and Bcl-2 expression. Furthermore, mYGJ induces HSCs apoptosis via ROS production and calcium release leading to intrinsic caspase-3 activation. This study thereby provides new mechanistic insight for the pharmaceutical effects of mYGJ in the treatment of patients with liver fibrosis. 

Of the mYGJ components, *Astragalus membranaceus* has been shown to elicit antifibrotic effect through inhibition of collagen synthesis and HSCs proliferation [16]. Moreover, Carapax trionycis may cause a decrease in the number of activated HSC and the total number of HSC [17]. These findings are in accord with our data and support the view that the antifibrotic effect of mYGJ is mainly mediated through regulation of HSCs proliferation and activation. In contrast, Sho-saiko-to (TJ-9) inhibits the proliferation of HSCs by induction of cell cycle arrest at the G0/G1 phase [18] and changes the balance of MMPs/TIMPs [19]. These studies together imply that different recipes of traditional Chinese medicine play diverse roles in suppression of HSC activation and may have different efficacy in improving the clinical outcome of patients with liver fibrosis. 

At least two distinct cellular pathways leading to HSC apoptosis account for the antifibrotic activity of mYGJ. At first, excessive ROS generation is crucial to mYGJ-induced HSC apoptosis (Figure 2(a)). This notion is consistent with a previous study reporting a component of mYGJ, *Melia toosendan *Sieb, induces hepatocyte death by production of ROS [14]. At low/moderate concentrations, ROS elicits beneficial effects on several important physiological responses, such as oxygen sensing, angiogenesis, control of vascular tone, regulation of cell growth, differentiation, and migration, in defense against infectious agents and the induction of a mitogenic response [20, 21]. In contrast, excessive ROS production induces cellular damage, such as cause of DNA damage and oxidation of proteins, impairs mitochondrial respiration, and is implicated in cell death. In this study, we reveal that the antifibrotic effect of mYGJ is closely related to ROS generation leading to HSC apoptosis. 

On the other hand, mYGJ may stimulate an increase of intracellular calcium and activate ER stress and mitochondrial pathway leading to the induction of apoptosis. According to the data we present in this study, mYGJ may induce ER stress with the Bax in the ER membrane undergoing conformational alteration and permit calcium release. Calpain is a calcium-dependent cysteine protease which is activated by release of calcium and subsequently activates procaspase 12 or causes mitochondrial membrane potential loss leading to the release of cytochrome *c* [19, 22]. However, calpain was not significantly changed in HSCs under mYGJ treatment while activated caspase 12 was detected in this condition (Figure 3(d)). It is likely that caspase 12 is activated by a calpain-independent manner. In accord with this notion, caspase 12 has been shown to be activated by the upstream caspase 7 which is activated by caspase 9 [22]. Alternatively, Bax directly causes mitochondria to release cytochrome *c* and activate caspase 9 [23]. Besides, Mattson and Chan have shown that small amounts of cytochrome *c* released from mitochondria function in a positive feedback loop by binding to Inositol phosphate-3 receptor on the endoplasmic reticulum, triggering calcium release, and thus amplifying calcium-dependent apoptosis [24]. These molecular changes together induce HSC apoptosis and account for the therapeutic effects of mYGJ on HSC activation and liver fibrosis.

Until now, how mYGJ mediates ROS generation in HSC is not yet completely understood. In aerobic cells, the mitochondrial respiratory chain is the major cellular source for ROS. It originates as a byproduct of oxygen metabolism in the electron transport chain within the mitochondria. ROS are also generated by tightly regulated cellular enzymes such as NADPH oxidase [20]. Hence, mYGJ may sustain ROS production by modulation of mitochondrial respiratory chain activity or, alternatively, regulating NADPH oxidase activity. Although the detailed molecular mechanisms remain to be investigated further, our data reported in this study indicate that mYGJ regulates Bcl-2-asscociated mitochondria signaling and thereby supports the notion that mYGJ modulates mitochondrial respiratory chain and causes excessive ROS production leading to cell apoptosis. 

**Figure 4:**
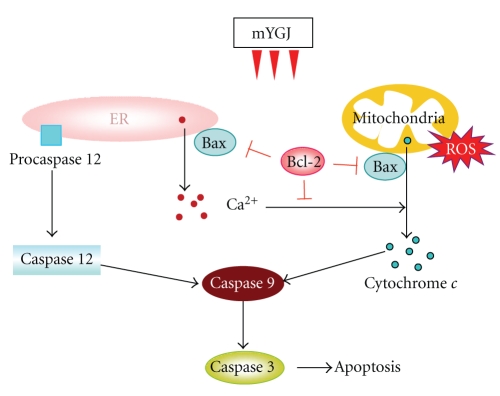
Hypothetical pathways by which mYGJ induces apoptosis of HSC-T6 cells. mYGJ promotes proapoptotic factor Bax expression, decreases antiapoptotic factor Bcl-2 expression, and along with the accumulation of ROS facilitates calcium and cytochrome c release to cytosol followed by activation of caspase 9 and caspase 3 leading to apoptosis. On the other hand, mYGJ may induce ER stress that results in an increase in cytosolic calcium concentration and activates-ER-associated caspase 12 subsequently joints the activation of caspase 9/ 3 cascade.

Together, our data support a model of action for mYGJ-mediated suppression of HSC activation as illustrated in Figure 4. In this notion, mYGJ induces the intrinsic pathway of apoptosis that involves Bcl-2-regulated mitochondria signaling, followed by enhancement of ROS accumulation and an increase in cytochrome *c* release from mitochondria to cytosol. On the other hand, mYGJ may induce ER stress that results in an increase in cytosolic calcium concentration and activation of caspase 12. Both pathways activate caspase 9/caspase 3 leading to activated HSC apoptosis. Whether mYGJ also elicits antifibrotic effect *in vivo* in the liver fibrosis animal model and is in accord with the mode of action we propose here is under investigation.

## 5. Conclusion

The mYGJ is a popular Chinese herbal medicine recipe and is widely administered to patients with chronic hepatitis and liver cirrhosis. Our results indicated that mYGJ inhibited the accumulation of activated HSCs and reduced HSC-activated marker *α*-SMA expression through an ROS-mediated intrinsic apoptosis pathway. Furthermore, ER stress-associated signalling may also be involved in the mYGJ-induced HSC apoptosis. We demonstrate for the first time the molecular basis for the antiliver fibrosis effect of mYGJ. This study may lead to new insight regarding how different traditional Chinese medicine recipes may be combined, according to their mechanisms of action, for treatment of liver fibrosis to elicit the maximal beneficial effects to the patients.

## Supplementary Material

Representative HPLC chromatograms of modified Yi Guan Jian. The Hitachi HPLC system equipped with a UV-VIS detector was used to analyze the extract of mYGJ. The amount of samples injected was 10 *μ*l of mYGJ extract which was dissolved in 10 ml of methanol and applied to ultrasound shock for 60 min. The 0.45 *μ*m membrane filtrated specimen was used to examine by HPLC. For Ferulic acid, the column was a 5 *μ*m Mightysil RP-18 GP (4.6 mm × 250 mm). The mobile phase was a gradient elution of 0.03% H3PO4 (A) and acetonitrile (B), commencing with 10% B for 10 min and then a linear gradient to 90% B was applied over 45 min. Flow rate was 1.0 ml/min. The detector was set at wavelength 320 nm. For Catalpol, the column was a Waters Cosmosil 5NH2-MS (4.6 mm× 250 mm).The mobile phase was eluted by acetonitrile over 75 min. Flow rate was 1.0 ml/min. The detector was set at wavelength 203 nm.

## Figures and Tables

**Table 1:** Composition of modified Yi Guan Jian.

Plant name	Family	Part used	Composition
Glehnia littoralis F.	Umbelliferae	Radix	3 (9.8%)
Ophiopogon japonicus (L. f.) Ker- Gawl.	Liliaceae	Radix	3 (9.8%)
Angelica sinensis (Oliv.) Diels.	Umbelliferae	Radix	3 (9.8%)
Rehmannia glutinosa Libosch.	Scrophulariaceae	Radix Rhizoma	6 (19.7%)
Lycium barbarum L.	Solanaceae	Fructus	3 (9.8%)
Melia toosendan Sieb. et Zucc.	Meliae	Fructus	1.5 (5%)
**Astragalus membranaceus (Fisch.) Bge.**	**Leguminosae**	**Radix**	**3 (9.8%)**
**Trionyx sinensis wiegmann.**	**Trionychidae**	**Shell**	**5 (16.4%)**
**Eupolyphaga sinensis walker.**	**Corydiidae**	**Dried body of female**	**3 (9.8%)**
			Total: 30.5 (100%)

Provided by Ko-Da pharmaceutical co. Taiwan.
