# Supplementary figures and images for: A Chinese Herbal Decoction, Modified Yi Guan Jian, Induces Apoptosis in Hepatic Stellate Cells through an ROS-Mediated Mitochondrial/Caspase Pathway
Source: Evid Based Complement Alternat Med. 2010 Sep 26;2011:459531. doi: 10.1155/2011/459531 (PMC2957151; doi:10.1155/2011/459531)

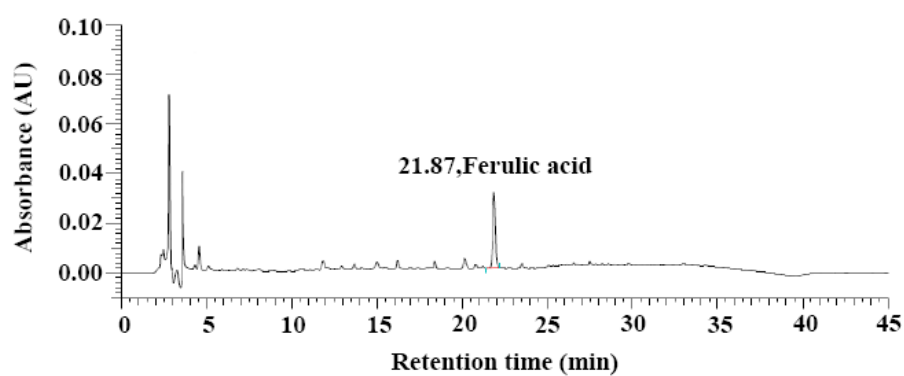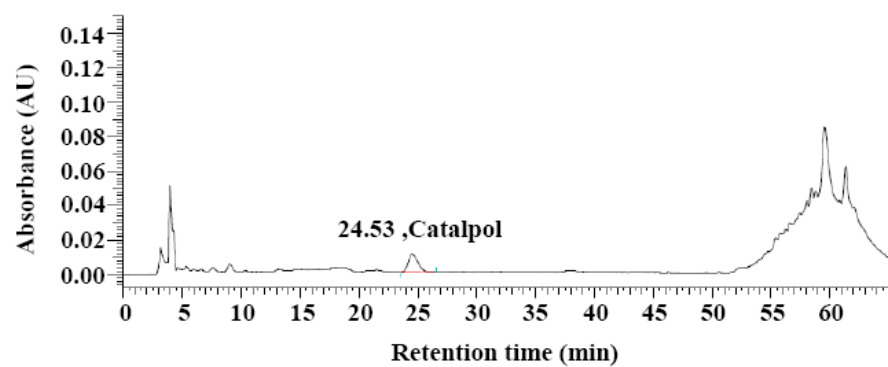

Supplemental Figure 1.  
Lin *et al.* ↑

Supplement: Supplementary file 1 — Representative HPLC chromatograms of modified Yi Guan Jian. The Hitachi HPLC system equipped with a UV-VIS detector was used to analyze the extract of mYGJ. The amount of samples injected was 10 μl of mYGJ extract which was dissolved in 10 ml of methanol and applied to ultrasound shock for 60 min. The 0.45 μm membrane filtrated specimen was used to examine by HPLC. For Ferulic acid, the column was a 5 μm Mightysil RP-18 GP (4.6 mm × 250 mm). The mobile phase was a gradient elution of 0.03% H3PO4 (A) and acetonitrile (B), commencing with 10% B for 10 min and then a linear gradient to 90% B was applied over 45 min. Flow rate was 1.0 ml/min. The detector was set at wavelength 320 nm. For Catalpol, the column was a Waters Cosmosil 5NH2-MS (4.6 mm× 250 mm).The mobile phase was eluted by acetonitrile over 75 min. Flow rate was 1.0 ml/min. The detector was set at wavelength 203 nm. [file 459531.f1.pdf]
